# Supplementary material for: Multi‐dataset identification of innovative feature genes and molecular mechanisms in keratoconus
Source: J Cell Mol Med. 2024 Sep 19;28(18):e70079. doi: 10.1111/jcmm.70079 (PMC11412914; doi:10.1111/jcmm.70079)
Supplement: Supplementary file 2 — Material S2. [file JCMM-28-e70079-s002.zip › 7.GSEA analysis/5.RNF217-AS1/KEGG_METABOLISM_OF_XENOBIOTICS_BY_CYTOCHROME_P450.html]

Details for gene set KEGG\_METABOLISM\_OF\_XENOBIOTICS\_BY\_CYTOCHROME\_P450[GSEA]

|  || Dataset | RNF217-AS1.group.cls#h\_versus\_l |
| Phenotype | group.cls#h\_versus\_l |
| Upregulated in class | h |
| GeneSet | KEGG\_METABOLISM\_OF\_XENOBIOTICS\_BY\_CYTOCHROME\_P450 |
| Enrichment Score (ES) | 0.17919993 |
| Normalized Enrichment Score (NES) | 0.57013756 |
| Nominal p-value | 1.0 |
| FDR q-value | 0.9926781 |
| FWER p-Value | 1.0 |
Table: GSEA Results Summary

  

Fig 1: Enrichment plot: KEGG\_METABOLISM\_OF\_XENOBIOTICS\_BY\_CYTOCHROME\_P450      
 Profile of the Running ES Score & Positions of GeneSet Members on the Rank Ordered List

  

| SYMBOL | TITLE | RANK IN GENE LIST | RANK METRIC SCORE | RUNNING ES | CORE ENRICHMENT || 1 | UGT1A8 | na | 901 | 0.455 | 0.0257 | Yes |
| 2 | CYP2C19 | na | 2150 | 0.337 | 0.0281 | Yes |
| 3 | UGT2B4 | na | 3115 | 0.286 | 0.0328 | Yes |
| 4 | CYP2C9 | na | 3299 | 0.279 | 0.0593 | Yes |
| 5 | ADH1B | na | 3511 | 0.271 | 0.0840 | Yes |
| 6 | CYP3A43 | na | 3651 | 0.265 | 0.1102 | Yes |
| 7 | UGT1A6 | na | 4124 | 0.248 | 0.1248 | Yes |
| 8 | ADH6 | na | 4165 | 0.247 | 0.1517 | Yes |
| 9 | ADH4 | na | 4223 | 0.244 | 0.1778 | Yes |
| 10 | UGT2B11 | na | 5963 | 0.184 | 0.1487 | Yes |
| 11 | GSTA2 | na | 6075 | 0.181 | 0.1660 | Yes |
| 12 | CYP1A1 | na | 6603 | 0.165 | 0.1696 | Yes |
| 13 | UGT2B28 | na | 7074 | 0.151 | 0.1732 | Yes |
| 14 | CYP1A2 | na | 7751 | 0.134 | 0.1689 | Yes |
| 15 | CYP2C18 | na | 7908 | 0.130 | 0.1792 | Yes |
| 16 | CYP3A4 | na | 9231 | 0.100 | 0.1525 | No |
| 17 | UGT2B10 | na | 9434 | 0.097 | 0.1577 | No |
| 18 | UGT2A3 | na | 10034 | 0.086 | 0.1502 | No |
| 19 | DHDH | na | 10141 | 0.084 | 0.1567 | No |
| 20 | GSTA5 | na | 10238 | 0.082 | 0.1633 | No |
| 21 | GSTM5 | na | 10401 | 0.080 | 0.1676 | No |
| 22 | CYP2B6 | na | 12406 | 0.050 | 0.1155 | No |
| 23 | CYP2C8 | na | 12822 | 0.045 | 0.1086 | No |
| 24 | CYP2E1 | na | 13283 | 0.039 | 0.0998 | No |
| 25 | GSTT1 | na | 14076 | 0.030 | 0.0803 | No |
| 26 | UGT2A1 | na | 14187 | 0.028 | 0.0804 | No |
| 27 | AKR1C1 | na | 15114 | 0.018 | 0.0557 | No |
| 28 | GSTZ1 | na | 15858 | 0.010 | 0.0355 | No |
| 29 | ADH7 | na | 16853 | 0.000 | 0.0068 | No |
| 30 | GSTO1 | na | 16930 | -0.001 | 0.0047 | No |
| 31 | GSTK1 | na | 17626 | -0.007 | -0.0145 | No |
| 32 | MGST3 | na | 17888 | -0.010 | -0.0209 | No |
| 33 | GSTP1 | na | 18119 | -0.012 | -0.0262 | No |
| 34 | MGST2 | na | 18715 | -0.018 | -0.0414 | No |
| 35 | GSTO2 | na | 21002 | -0.040 | -0.1028 | No |
| 36 | AKR1C4 | na | 22318 | -0.054 | -0.1346 | No |
| 37 | GSTM3 | na | 22649 | -0.058 | -0.1375 | No |
| 38 | ALDH3B2 | na | 23124 | -0.063 | -0.1440 | No |
| 39 | ALDH3A1 | na | 23827 | -0.073 | -0.1560 | No |
| 40 | CYP3A7 | na | 24059 | -0.076 | -0.1539 | No |
| 41 | ALDH3B1 | na | 24098 | -0.077 | -0.1463 | No |
| 42 | ADH1A | na | 24873 | -0.088 | -0.1586 | No |
| 43 | GSTA3 | na | 25136 | -0.093 | -0.1556 | No |
| 44 | GSTT2 | na | 25629 | -0.100 | -0.1584 | No |
| 45 | GSTM1 | na | 25692 | -0.101 | -0.1486 | No |
| 46 | GSTA4 | na | 25928 | -0.106 | -0.1434 | No |
| 47 | ALDH1A3 | na | 26655 | -0.119 | -0.1508 | No |
| 48 | UGT2B7 | na | 26756 | -0.121 | -0.1399 | No |
| 49 | UGT2B15 | na | 26916 | -0.124 | -0.1304 | No |
| 50 | CYP1B1 | na | 27274 | -0.132 | -0.1257 | No |
| 51 | CYP2S1 | na | 28056 | -0.150 | -0.1312 | No |
| 52 | ADH5 | na | 28827 | -0.171 | -0.1339 | No |
| 53 | GSTM2 | na | 28859 | -0.172 | -0.1152 | No |
| 54 | EPHX1 | na | 29424 | -0.189 | -0.1100 | No |
| 55 | GSTM4 | na | 29778 | -0.199 | -0.0975 | No |
| 56 | AKR1C3 | na | 29929 | -0.204 | -0.0786 | No |
| 57 | CYP3A5 | na | 33128 | -0.370 | -0.1288 | No |
| 58 | ADH1C | na | 33785 | -0.445 | -0.0970 | No |
| 59 | CYP2F1 | na | 34131 | -0.509 | -0.0490 | No |
| 60 | MGST1 | na | 34361 | -0.582 | 0.0106 | No |
Table: GSEA details [plain text format]

  

Fig 2: KEGG\_METABOLISM\_OF\_XENOBIOTICS\_BY\_CYTOCHROME\_P450      
 Blue-Pink O' Gram in the Space of the Analyzed GeneSet

  

Fig 3: KEGG\_METABOLISM\_OF\_XENOBIOTICS\_BY\_CYTOCHROME\_P450: Random ES distribution      
 Gene set null distribution of ES for **KEGG\_METABOLISM\_OF\_XENOBIOTICS\_BY\_CYTOCHROME\_P450**
